# Activating *JAK1* mutation may predict the sensitivity of JAK-STAT inhibition in hepatocellular carcinoma

**DOI:** 10.18632/oncotarget.6684

**Published:** 2015-12-19

**Authors:** Shuqun Yang, Chonglin Luo, Qingyang Gu, Qiang Xu, Guan Wang, Hongye Sun, Ziliang Qian, Yexiong Tan, Yuxin Qin, Yuhong Shen, Xiaowei Xu, Shu-Hui Chen, Chi-Chung Chan, Hongyang Wang, Mao Mao, Douglas D. Fang

**Affiliations:** ^1^ Oncology Business Unit, WuXi AppTec Co., Ltd., Waigaoqiao Free Trade Zone, Shanghai, China; ^2^ Genome Center, WuXi AppTec Co., Ltd., Waigaoqiao Free Trade Zone, Shanghai, China; ^3^ Shanghai Johnson & Johnson Pharmaceuticals Ltd., Shanghai, China; ^4^ Eastern Hepatobiliary Surgery Hospital/Institute of Shanghai, Shanghai, China; ^5^ Pathology and Laboratory Medicine, Hospital of the University of Pennsylvania, Philadelphia, PA, USA; ^6^ Domestic Discovery Service Unit, WuXi AppTec Co., Ltd., Waigaoqiao Free Trade Zone, Shanghai, China; ^7^ Translational Bioscience and Diagnostics, WuXi AppTec Co., Ltd., Waigaoqiao Free Trade Zone, Shanghai, China; ^8^ Current address: Cancer Translational Research, China Novartis Institute for Biomedical Research, Shanghai, China

**Keywords:** HCC, PDX, JAK1, ruxolitinib

## Abstract

Hepatocellular carcinoma (HCC) is the fifth most common type of cancers worldwide. However, current therapeutic approaches for this epidemic disease are limited, and its 5-year survival rate hasn't been improved in the past decades. Patient-derived xenograft (PDX) tumor models have become an excellent *in vivo* system for understanding of disease biology and drug discovery. In order to identify new therapeutic targets for HCC, whole-exome sequencing (WES) was performed on more than 60 HCC PDX models. Among them, four models exhibited protein-altering mutations in *JAK1* (Janus Kinase 1) gene. To explore the transforming capability, these mutations were then introduced into HEK293FT and Ba/F3 cells. The results demonstrated that *JAK1^S703I^* mutation was able to activate JAK-STAT (Signal Transducer and Activator of Transcription) signaling pathway and drive cell proliferation in the absence of cytokine stimulation *in vitro*. Furthermore, the sensitivity to the treatment of a JAK1/2 inhibitor, ruxolitinib, was observed in *JAK1^S703I^* mutant PDX model, but not in other non-activating mutant or wild type models. Pharmacodynamic analysis showed that phosphorylation of STAT3 in the Ruxolitinib-treated tumor tissues was significantly suppressed. Collectively, our results suggested that *JAK1^S703I^* is an activating mutation for JAK-STAT signaling pathway *in vitro* and *in vivo*, and JAK-STAT pathway might represent a new therapeutic approach for HCC treatment. Monotherapy using a more potent and specific JAK1 inhibitor and combinatory therapy should be further explored in *JAK1* mutant PDX models.

## INTRODUCTION

Hepatocellular carcinoma (HCC) is the fifth most common cancer type around the world. The incidence of HCC remains particularly high in East Asia and sub-Saharan Africa [1], and has been rising in North American and Western Europe [2, 3]. Its epidemics correlates with the prevalence of hepatitis B and C virus in Asia and U.S./Europe, respectively [4, 5]. In fact, chronic viral infections account for more than 70% cases of HCC globally.

The prognosis for patient with advanced HCC remains poor. Although sorafenib has been approved for patients with advanced HCC in 2007 [6], the benefits with sorafenib are modest. In the past years, there have been renewed and continued interests in identifying novel molecular targets in HCC.

Mechanistically, alterations of multiple signaling pathways were detected in HCC patients, including WNT, FGFR, VEGFR, and PDGFR. Among them, deregulation of WNT/beta-catenin signaling was found in 40-70% HCC patients, which is responsible for cell proliferation, migration, and self-renewal. Currently, pharmacological inhibition of WNT pathway is being tested at early stage of clinical trials. Further, activation of FGFR, VEGFR, and PDGFR signaling pathways is closely related to angiogenesis, a key process during HCC carcinogenesis. Therefore, several multi-kinase inhibitors have been evaluated for HCC patients, including sunitinib and dovitinib.

Another important signaling pathway for HCC is JAK-STAT pathway, which is critical to transferring extracellular signal into nucleus for transcriptional regulation. JAK-STAT pathway resides at downstream of various transmembrane receptors, including cytokine receptors such as interferon (IFN) and interleukin 6 (IL-6), as well as aforementioned growth-factor receptors, FGFR, VEGFR, and PDGFR. Once activated, Janus Kinase (JAK) family, JAK1, JAK2, JAK3, and TYK2, could phosphorylate the cytoplasmic portion of cytokine receptors, which in turn recruit signal transducer and activator of transcription (STAT) *via* its SH2 domain. Dimerization of STATs then occurs when they are associated with tyrosine kinase receptors, leading to their translocation into nucleus and increased transcription of downstream genes, such as c-MYC, CCND1, and VEGF. Therefore, functional JAK-STAT pathway is required for proliferation and survival of normal cells. [7–9]

During the carcinogenesis, several cytokine and growth factor receptor kinases are constitutively activated by different mechanisms. As a result, JAK-STAT pathway is essential for the uncontrolled growth of cancer cells, angiogenesis and metastasis. Several *JAK1* mutations were found in different cancer types, such as leukemia, breast cancer, lung cancer, and HCC. *JAK1^V658F^* was found in leukemia patients, leading to constitutional activation of *JAK1* [10, 11]. Further, seven distinct protein-altering *JAK1* mutations were previously identified in tumors from HCC patients by whole-genome sequencing (WGS). Moreover, both of *JAK1^S729C^* and *JAK1^S703I^* mutations were recurrent and proved to be activating mutations *in vitro* [12]. On the other hand, a point mutation of *JAK2^V617F^*, was found in polycythemia vera (PV), essential thrombocythemia (ET), and primary myelofibrosis (PMF) patients [13–15]. This mutation causes the constitutive activation of JAK2 kinase *via* disrupting its auto-inhibition.

In the wake of strong correlation between *JAK2^V617F^* mutation and myeloproliferative neoplasms (MPN), the search for JAK inhibitors has been accelerated. Multiple molecules targeting different members of JAK kinase family have been synthesized and characterized. Among them, ruxolitinib was approved by FDA for patients with MPN. According to the results of two phase III clinical trials for myelofibrosis (COMFORT-I and COMFORT-II), ruxolitinib could alleviate the splenomegaly and other symptoms for 30-40% of patients [16, 17]. Mechanistically, ruxolitinib targets both JAK1 and JAK2 with similar IC50 by competitive inhibition of these two kinases [18]. The IC_50_ of ruxolitinib for JAK1 and JAK2 *in vitro* were 3.3 nM and 2.8 nM, respectively [19]. In the preclinical study, ruxolitinib could effectively inhibit the proliferation of *JAK2^V617F^*-positive cell line and alleviate MPN symptoms in *JAK2^V617F^* transgenic mice model [19]. However, the effect of ruxolitinib has not been extensively studied in solid tumors.

In the present study, we aimed to identify novel therapeutic targets in HCC and discovered four *JAK1* mutations in HCC PDX models through WES. Their identities were confirmed by targeted sequencing, and they were then characterized for activation of JAK-STAT pathway and oncogenic potential *via* Western blot analysis and *in vitro* proliferation assay, respectively. Moreover, *in vivo* efficacy studies of ruxolitinib were conducted in *JAK1*-mutant PDX models. Taken together, our data suggest that activating *JAK1* mutations may be molecular targets for the treatment of HCC.

## RESULTS

### Identification of *JAK1* mutations in HCC PDX models

More than 160 HCC PDX models were established at WuXi AppTec in the past three years, of which over 60 models were characterized by WES. Among them, four models (LI-03-0012, LI-03-0155, LI-03-0191, and LI-03-0257) were identified with non-synonymous mutations in *JAK1* gene. These *JAK1* mutations, including N451S in LI-03-0155, E483D in LI-03-0257, S703I in LI-03-0191, and A1086S in LI-03-0012 models, were then verified by Sanger sequencing with targeted primers (Data not shown).

**Figure 1:**
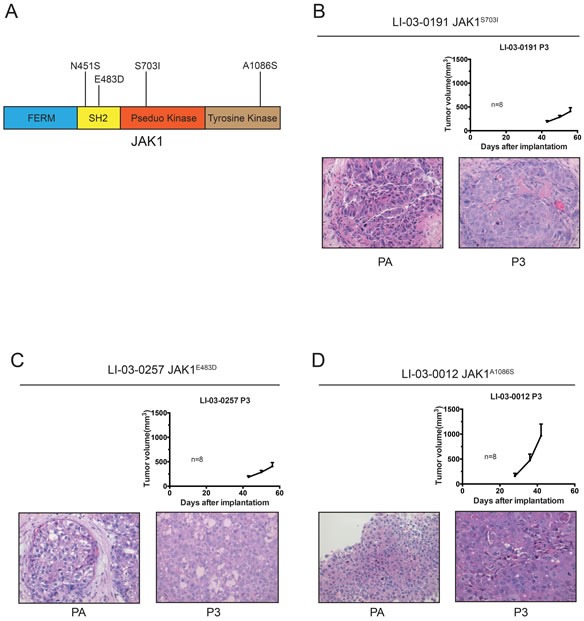
Growth curves and H&E staining of three *JAK1*-mutated PDX models **A.** Diagram of JAK1 protein, illustrating non-synonymous mutations discovered in HCC PDX models. **B.-D.** Tumor growth potentials were measured in three *JAK1*-mutant PDX models (P3). Data were shown as mean ± SEM. The H&E staining sections of patient tumor (PA) and PDX tissues at P3 were shown (magnification, 40X).

Specifically, S703I mutation was found in the pseudo-kinase domain of JAK1 protein, and could potentially cause the disruption of auto-inhibition of JAK1 kinase. Notably, S703I was previously identified in tumors of two HCC patients, and proved to be an activating mutation of *JAK1* gene [12]. For the other three *JAK1* mutations, A1086S is located in catalytic kinase domain, whereas N451S and E483D are in the SH2 domain of JAK1 protein. (Figure 1A)

All four PDX models have been passaged in BALB/c nude mice for up to 3 generations. And their growth kinetics did not change much during this process (Data not shown). The growth rate of LI-03-0012 is noticeably faster than rest models. We suspected that other oncogenic mutations may contributed to this. In addition, H&E staining confirmed that pathological features were maintained in each PDX model, comparing to the patient's tumor (PA) (Figure 1B, 1C, and 1D).

Taken together, mutations of *JAK1* gene were identified in four HCC PDX models *via* WES, and validated by Sanger sequencing. These *JAK1*-mutant PDX models were established and ready for pharmacological studies.

### Characterization of four *JAK1* mutations

**Figure 2:**
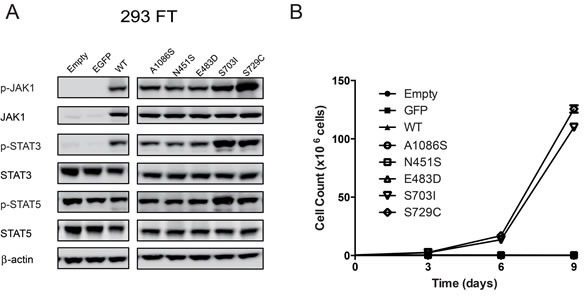
Characterization of four *JAK1* mutations **A.** 293FT cells were transiently transfected with pLVX-IRES-Neo plasmids containing open reading frames (ORFs) of wild-type and five mutant forms of *JAK1* (*JAK1^N451S^*, *JAK1^E483D^*, *JAK1^S703I^*, *JAK1^A1086S^*, and *JAK1^S729C^*), *EGFP*, and empty vector control. The expression levels of phosphorylated and total JAK1, STAT3, and STAT5 were evaluated by western blot analyses. Beta-actin served as a loading control. The experiment was performed twice. The representative results were shown. **B.** Ba/F3 cells stably infected with lentivirus expressing wild-type *JAK1*, the five mutants, *EGFP*, and empty vector control were cultured in the absence of IL3. The cell number of each line were counted every three days and plotted against time. The experiment was performed twice with triplicates. The representative results were shown.

To explore the biological functions of *JAK1* mutations in JAK-STAT signaling pathway, we introduced these mutations into pLVX-IRES-Neo-JAK1 plasmid. Plasmids containing *EGFP*, wild-type *JAK1*, *JAK1^N451S^*, *JAK1^E483D^*, *JAK1^S703I^*, *JAK1^A1086S^*, and *JAK1^S729C^* were constructed. The latter is a known and recurrent activating mutation of *JAK1* [12]. Then, plasmids containing distinct *JAK1* genes were transiently transfected into HEK293FT cells. The expression levels of phosphorylated JAK1, total JAK1, phosphorylated STAT3, total STAT3, phosphorylated STAT5, and total STAT5 were analyzed by Western blot, while β-actin served as a loading control. Notably, similar to the positive control *JAK1^S729C^* [12], ectopic expression of *JAK1^S703I^* in HEK293FT cells resulted in elevated expression levels of p-JAK1, p-STAT3, and p-STAT5 (Figure 2A).

To further evaluate the transformation ability of these *JAK1* mutations, Ba/F3 cells were stably infected with lentivirus expressing EGFP, wild-type *JAK1*, *JAK1^N451S^*, *JAK1^E483D^*, *JAK1^S703I^*, *JAK1^A1086S^*, and *JAK1^S729C^*, respectively. In the absence of IL-3, only the cells expressing *JAK1^S703I^* or positive control *JAK1^S729C^* were capable of continual proliferation, whereas the cells expressing the *EGFP*, wild-type and the other mutations of *JAK1* gene ceased to grow *in vitro* (Figure 2B). In addition, no other obvious driver mutations for HCC, such as *CTNNB1*, have been found in genome of LI-03-0191 PDX model. These data suggest that, among all of four JAK1 mutations identified in the PDX models, only *JAK1^S703I^* mutation is able to activate JAK-STAT signaling pathway and exhibits a transforming potential.

### Anti-tumor activity of ruxolitinib in *JAK1^S703I^*-mutant PDX model

**Figure 3:**
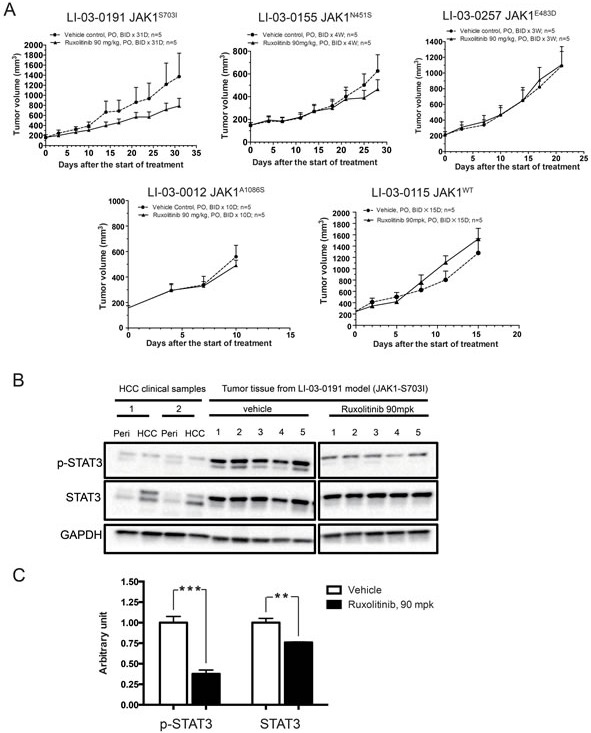
Anti-tumor efficacy of ruxolitinib in *JAK1*-mutant and *JAK1*-WT PDX models **A.** BALB/c nude mice bearing *JAK1*-mutant or *JAK1*-WT PDX tumors were administered with either vehicle (•) or ruxolitinib (90 mpk, BID) (▴) for an indicated length of time. Tumor volumes were plotted against time. **B.** PDX tumor tissues of *JAK1^S703I^*-mutant model were harvested from animals of vehicle and ruxolitinib-treated group from the efficacy study. The expression levels of phosphorylated STAT3, total STAT3, and GAPDH in these PDX tumor tissues, as well as tumor and adjacent normal tissues from clinical HCC patients, were assessed by western blot analyses. The experiment was performed twice and the representative results were shown. **C.** Quantification of expression levels of phosphorylated STAT3 and total STAT3 in PDX tumor tissues from vehicle and ruxolitinib-treated group of *JAK1^S703I^*-mutant model (***, *p* < 0.001; **, *P* < 0.01).

*In vivo* efficacy studies of JAK1/2 inhibitor, ruxolitinib, were conducted in these four *JAK1*-mutant models and a *JAK1*-WT PDX model as a control (Figure 3A). The results showed that, only in LI-03-0191 model bearing *JAK1^S703I^* mutation, treatment with ruxolitinib resulted in a tumor growth inhibition (TGI, 48%) in comparison with the vehicle control group. No significant anti-tumor activity of ruxolitinib was observed in rest three *JAK1*-mutant models, as well as *JAK1*-WT model. These results indicate that tumor growth in LI-03-0191 PDX model is sensitive to inhibition of JAK kinase, suggesting that *JAK1^S703I^* may play a critical role for tumorigenesis in this HCC model.

### Pharmacodynamics analysis of JAK-STAT signaling pathway in tumor tissues

To further investigate the molecular mechanisms behind ruxolitinib-induced growth inhibition, tumor tissues were harvested from LI-03-0191 model in the above efficacy study. Constitutively activated STAT3 per se has been shown to be able to function as an oncogene, and STAT3 has been widely accepted as a cancer therapeutic target [20–22]. Thus, the expression levels of phosphorylated and total STAT3 in these samples were analyzed by Western blot, in comparison with the tumor samples obtained from two HCC patients.

Firstly, the level of total STAT3 in two HCC patient samples appeared to be higher than that in corresponding adjacent normal tissues. Interestingly, treatment of ruxolitinib led to a significant reduction (50%) of the phosphorylation level of STAT3 in tumors collected from all five animals in LI-03-0191 model, compared with that in vehicle group. In addition, a modest reduction of total STAT3 level was also observed in ruxolitinib-treated group in comparison with vehicle group (Figure 3B and 3C). These results suggest that ruxolitinib may cause the suppression of JAK-STAT signaling pathway in PDX models bearing *JAK1^S703I^* mutation.

## DISCUSSION

In this study, we have characterized four *JAK1* mutations in over 60 HCC PDX models established at WuXi AppTec. These mutations were identified by WES analysis, confirmed by Sanger sequencing, and then evaluated for their transforming potential *in vitro*. Among them, *JAK1^S703I^* was able to activate JAK-STAT signaling pathway and drove *in vitro* cell proliferation independent of IL-3 stimulation in otherwise IL-3 dependent Ba/F3 cell line. Further, the tumor growth of HCC PDX model bearing *JAK1^S703I^* mutation was sensitive to treatment of a JAK1/2 inhibitor, ruxolitinib. These data indicated that *JAK1^S703I^* may be an activating mutation in HCC.

Currently, multi-kinase inhibitor sorafenib is used as first line therapy for unresectable advanced HCC patients, which only yields limited elongation in patients’ survival time [23]. Therefore, the search for new therapeutic targets in HCC is urgently needed.

Previous studies have demonstrated that pharmacological inhibition of STAT protein and upregulation of Src-homology protein tyrosine phosphatases (SHPs), a negative regulator of JAK-STAT pathway, could prevent growth of HCC cell line *in vitro* and *in vivo* [24–26]. In addition, an earlier study reported that targeting JAK-STAT pathway in HCC cell lines by ruxolitinib led to apoptosis *in vitro* [27]. These evidences strongly suggested that abnormal activation of JAK-STAT pathway is associated with HCC. However, since most studies were conducted *in vitro* or in cell line derived xenograft models, it is hard to directly translate these findings to clinical development of HCC therapies. In addition, though ruxolitinib was approved by FDA for myelofibrosis in 2007, it is not clear whether ruxolitinib is able to suppress *in vivo* growth of tumors with aberrant JAK-STAT signaling pathway.

Intriguingly, as shown in our study, inhibition of activated JAK-STAT signaling pathway caused by *JAK1^S703I^* mutation produced an obvious delay of tumor progression in HCC PDX models. PDX models prove to be biologically stable, which is superior to cancer-cell-derived xenograft model [28]. HCC PDX models can also maintain the tumor microenvironment and vascular structure of patient tumors. Given that JAK-STAT signaling pathway has been demonstrated to play an important role in tumor microenvironment [29], our results in PDX models will shed light on the clinical development of HCC therapies. Further, these results demonstrated that ruxolitinib is capable of shrinking solid tumors, such as HCC, with activating JAK1 mutation.

In addition, since JAK-STAT pathway is important for various physiological functions, only HCC patients with upregulated JAK-STAT signaling could be treated with JAK inhibitors, thus a therapeutic window may exist. And accurate detection of mutations in related cancer genes is also critical before treatment strategy can be finalized for each individual patient.

Admittedly, further investigations are warranted to answer whether other activating mechanisms exist for JAK-STAT pathway in HCC. Nevertheless, the present study suggests that, in addition to standard-of-care therapy, a portion of HCC patients could benefit from targeting JAK-STAT signaling pathway. Combinational or sequential therapies based on sorafenib and JAK inhibitors would likely provide larger therapeutic margin to HCC patients with activated JAK-STAT pathway.

## MATERIALS AND METHODS

### Establishment of HCC PDX Models

In compliance with the protocol approved by the Institutional Review Board of Eastern Hepatobiliary Surgery Hospital/Institute of Shanghai and with the subject's informed consent, primary tumor tissues were collected for PDX establishment as described earlier [30]. In brief, surgically resected primary tumor tissues (designated as PA) were implanted subcutaneously in female BALB/c nude mice (Shanghai SLAC Laboratory Animal Co., Ltd., Shanghai, China). Once the first generation of xenograft (designed as P0) was established, serial implantations in BALB/c nude mice were performed to expand the xenograft tumors (i.e., P1, P2, P3, and beyond). Tumor volume was calculated as 0.5 × length × width^2^, and plotted against time to generate growth curves. All procedures and protocols were approved by the Institutional Animal Care and Use Committee of WuXi AppTec.

### Histology

Pieces of patient samples (PA) or PDX tissues at each passage were collected and stained with hematoxylin and eosin [30]. Histopathology was reviewed by a board-certified pathologist (X.X.).

### Tissue processing for genomic studies

Genomic DNA was isolated using a QIAamp DNA mini kit (Qiagen, Germantown, MD). The concentrations were quantified using NanoDrop ND-1000 spectrophotometer (NanoDrop, Wilmington, DE). DNA samples with A_260/280_ ratios between 1.8 and 2.0 and A_260/230_ ratios above 2.0, and proven to be high quality by gel electrophoresis were used for WES and SNP 6.0 array analyses.

### Whole exome sequencing (WES)

One microgram of each DNA sample isolated from P3 xenograft tumors was used for library construction using a TruSeq DNA sample preparation kit (Illumina, San Diego, CA). Libraries were pooled (500 ng each) for exome capture and amplification using the TruSeq Exome Enrichment kit (Illumina). Sequencing was then performed with paired-end 2×100 base reads on the HiSeq 2000 platform (Illumina). Raw FASTQ files were first processed by a proprietary algorithm to filter out mouse sequence contaminations. We have shown that this filter step does not affect the human SNP detection [31, 32]. After mouse sequence removed, sequencing reads were aligned to human reference genome hg19/GRCh37 by BWA 0.6.1 and processed to variants calling by GATK 1.6.

### Reverse transcription polymerase chain reaction and direct sequencing

Total RNA (1 μg) was reversely transcribed into single-stranded cDNAs using High Capacity cDNA Reverse Transcription Kits (Life Technologies, Carlsbad, CA) following the manufacturer's instructions. One microliter of cDNA was used for a subsequent 25-μl polymerase chain reaction (PCR) amplification using a Premix Prime STAR HS PCR amplification kit (Takara Bio, Otsu, Shiga, Japan). To detect *JAK1* mutations, we designed the forward and reverse primers targeting the sequences flanking the mutation points detected by whole-exome sequencing. PCR primers: LI-03-0257-Forward TCGGTATTTCCGCTACGCTC; LI-03-0257-Reverse ACACCTCATGGCTGTATGGC; LI-03-0191-Forward CCTTGTGACATTTAGCCGCC; LI-03-0191-Reverse TAAGCACCAGGCACACCTTT; LI-03-0012-Forward AGATACGTGCTACCGAGAGC; LI-03-0012-Reverse AACCAAGCAGAGGGATGGAC; LI-03-0257-Forward CGGTATTTCCGCTACGCTCA; LI-03-0257-Reverse CAGGCTACACTGGACACCTC.

### Cell culture

All cells were cultured in a humidified incubator with 5% CO_2_ at 37°C. HEK293FT cells were maintained in DMEM medium (high glucose) supplemented with 10% FBS, 0.1mM nonessential amino acids (Invitrogen), 6 mM L-Glutamine and 1 mM Sodium Pyruvate. Ba/F3 cells were maintained in RPMI1640 medium containing 10% FBS and 2.5 ng/ml IL-3 (Invitrogen)

### Plasmids construction, infection and transformation assay

Human *JAK1* open reading frames (ORFs) for wild-type and mutant variants (A1086S, N451S, E483D, S703I and S729C) as well as an EGFP ORF were synthesized and constructed into the lentiviral vector pLVX-IRES-Neo (Clontech, Mountain View, CA). Lentivirus was packaged using HEK293T cells and concentrated using polyethylene glycol (Sigma, St. Louis, MO). Ba/F3 cells cultured in RPMI1640 medium containing 10% FBS and 2.5 ng/ml IL-3 were infected with viruses carrying *JAK1* ORFs (wild-type or mutant) or EGFP as a control followed by G418 (Invitrogen) selection and subsequent expansion. For assaying transforming potential of *JAK1* mutants, transduced Ba/F3 cells were cultured in the absence of IL-3 in triplicates. Cell counting was performed every 3 days for a total of 9 days.

### Transfection and western blot analysis

HEK293FT cells were seeded the day before at 8×10^5^ cells/well in a 6-well plate, and transfected with 2.5 ug the fore-mentioned lentiviral plasmid DNA (*JAK1* wild-type, *JAK1* mutants or EGFP) using Lipofectamine 2000 (Invitrogen). 48 h post-transfection, cells were serum starved for 4 hr and then lysed in RIPA buffer containing protease inhibitors (Roche, Basel, Swiss) and phosphatase inhibitors (Sigma). Protein lysates were then diluted by 4X LDS Sample Buffer and 10X Reducing Agent, denatured by boiling, and separated by SDS-PAGE. Western blotting was performed as described earlier [33]. Following antibodies from Cell Signaling Technology (Danvers, MA) were used: phosphorylated JAK1 (p-JAK1; Tyr1022/1023), JAK1, phosphorylated STAT3 (p-STAT3; Tyr705), STAT3α, phosphorylated STAT5 (p-STAT5; Tyr694), STAT5 and GAPDH. The antibody for β-actin was purchased from Sigma.

### *In vivo* efficacy study

Ruxolitinib was synthesized and formulated in 5% DMSO, 0.5% methylcellulose and 0.1% Tween 80. Tumor tissues were cut into small fragments of approximately 30 mm^3^ under sterile conditions. BALB/c nude mice were implanted subcutaneously with a tumor fragment by using a trocar. When the average tumor size reached 150 to 200 mm^3^, tumor size-matched mice were randomly assigned to two groups with five mice in each group. The tumor-bearing mice were given ruxolitinib at 90 mg/kg twice per day orally [34], or vehicle alone for indicated time. Tumor volumes and body weights were measured using calipers twice a week.
